# Texture analysis of CT colonography to develop a novel imaging biomarker for the management of colorectal cancer

**DOI:** 10.1002/ags3.12852

**Published:** 2024-08-26

**Authors:** Hisashi Mamiya, Toru Tochigi, Koichi Hayano, Gaku Ohira, Shunsuke Imanishi, Tetsuro Maruyama, Yoshihiro Kurata, Yumiko Takahashi, Atsushi Hirata, Hisahiro Matsubara

**Affiliations:** ^1^ Department of Frontier Surgery Chiba University Graduate School of Medicine Chiba Japan

**Keywords:** biomarker, colorectal cancer, CT colonography, gray level co‐occurrence matrix, texture analysis

## Abstract

**Background:**

Recent studies have focused on evaluating the biomarker value of textural features in radiological images. Our study investigated whether or not a texture analysis of computed tomographic colonography (CTC) images could be a novel biomarker for colorectal cancer (CRC).

**Methods:**

This retrospective study investigated 263 patients with CRC who underwent contrast‐enhanced CTC (CE‐CTC) before curative surgery between January 2014 and December 2017. Multiple texture analyses (fractal, histogram, and gray‐level co‐occurrence matrix [GLCM] texture analyses) were applied to CE‐CTC (portal‐venous phase), and fractal dimension (FD), skewness, kurtosis, entropy, and GLCM texture parameters, including GLCM‐correlation, GLCM‐autocorrelation, GLCM‐entropy, and GLCM‐homogeneity, of the tumor were calculated. These texture parameters were compared with pathological factors (tumor depth, lymph node metastasis, vascular invasion, and lymphatic invasion) and overall survival (OS).

**Results:**

Tumor depth was significantly associated with FD, kurtosis, entropy, GLCM‐correlation, GLCM‐autocorrelation, GLCM‐entropy, and GLCM‐homogeneity (*p* = 0.001, 0.001, 0.001, 0.001, 0.018, 0.008, and 0.001, respectively); lymph node metastasis was associated with GLCM‐homogeneity (*p* = 0.004); lymphatic invasion was associated with GLCM‐correlation and GLCM‐homogeneity (*p* = 0.001 and 0.012, respectively); and venous invasion was associated with FD, entropy, GLCM‐correlation, GLCM‐autocorrelation, and GLCM‐entropy of the tumor (*p* = 0.001, 0.033, 0.021, 0.046, respectively). In the Kaplan–Meier analysis, patients with high GLCM‐correlation tumors or high GLCM‐homogeneity tumors showed a significantly worse OS than others (*p* = 0.001 and 0.04, respectively). Multivariate analyses showed that the GLCM correlation was an independent prognostic factor for the OS (*p* = 0.021).

**Conclusion:**

CE‐CTC‐derived texture parameters may be clinically useful biomarkers for managing CRC patients.

## Graphical abstract

GLCM derived from texture analyses using CE‐CTC images may become a potentially viable biomarker for assessing the tumor malignancy of CRC before surgery.

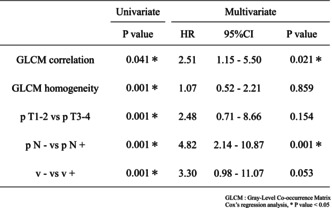

## 1 INTRODUCTION

Colorectal cancer (CRC) is the third most common malignancy and second leading cause of cancer‐related deaths worldwide. An estimated 1.8 million patients were newly diagnosed worldwide in 2018, and approximately 880 000 died due to CRC. The 5‐year survival rate for patients with stage I CRC is over 90%, while that for patients with stage IV CRC is only 11%.^1^ Therefore, the early detection and diagnosis of CRC are directly related to its prognosis, and an accurate evaluation and prediction of the prognosis, including malignancy and risk of recurrence, is important for selecting the appropriate treatment for CRC.

However, there are few factors that are more effective and useful in evaluating the malignancy of CRC than clinicopathological factors, such as tumor differentiation, tumor depth, lymph node metastasis, distant metastasis, and vascular invasion.

It has been reported that most cancers generally have structural heterogeneity, which is recognized as an important issue leading to aggressiveness of cancer and chemotherapy.^2^ Thus, quantification of structural abnormality of the tumor has a potential to be a biomarker for cancer treatment. Recently, the studies have focused on using texture analyses of medical images to quantify the structural abnormality of tumor, and reported that textural features in medical images can be a biomarker for cancer treatment and prognosis.^3^

Computed tomographic colonography (CTC) is widely used for the preoperative examination of CRC, as it is minimally invasive, does not require skilled hands for colonoscopy or barium enema, and is highly reproducible.^4^

To our knowledge, no study has reported that structural abnormality of tumor measured on CTC images is associated with pathologic features and prognosis of CRC.

The present study investigated whether or not texture analyses of CRC using CTC images is useful for diagnosing CRC malignancy.

## 2 MATERIALS AND METHODS

This retrospective study was performed with the approval of the Institutional Review Board of Chiba University Graduate School of Medicine. All patients provided their written informed consent to undergo a contrast‐enhanced CTC (CE‐CTC) examination, but participation was not required because of the retrospective nature of this study.

**FIGURE 1:**
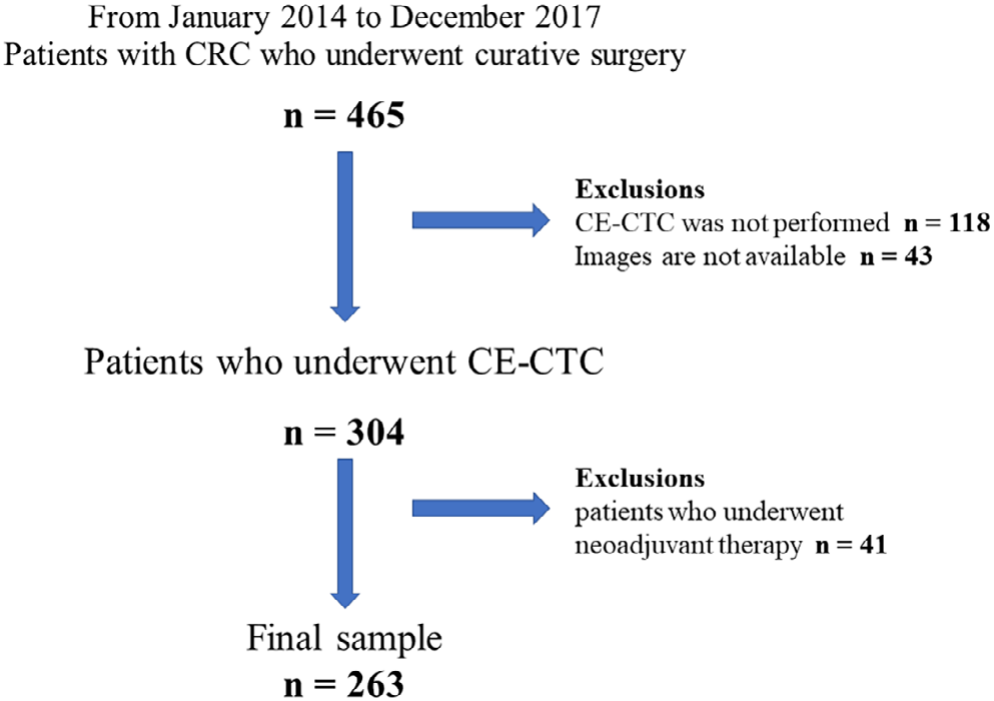
Flowchart of this study sample.

We retrospectively identified 465 patients with CRC who underwent CTC before curative surgery for CRC between January 2014 and December 2017. The following patients were excluded: (1) those who underwent only non‐CE‐CTC; (2) those for whom imaging analysis was not available in medical records; and (3) those who received neoadjuvant chemotherapy (NAC) or neoadjuvant chemoradiotherapy (NACRT) before surgery. In total, 263 patients were eligible for this study (Figure 1).

All patients in this study underwent preoperative colonoscopy.

All tumor locations and sizes were confirmed by colonoscopy and CTC images.

Height was not measured in this study.

### 2.1 CTC technique

The pre‐treatment for the CE‐CTC examination consisted of a change to a low‐residue diet the day before the examination, 100 mL of iodinated oral contrast agent after each meal, and 250 mL of magnesium citrate at 9 p.m.

Before the CTC examination, a rectal tube was inserted, and carbon dioxide gas was pumped into the rectum and colon at a constant pressure of 20 mmHg using a dedicated device (PROTOCO2L; BRACCO Imaging S.p.A., Milan, Italy). CTC was performed using a 64‐section multidetector row CT scanner (Revolution EVO; GE Healthcare, Milwaukee, WI, USA). Contrast medium (300 mgI/mL, 600 mgI/kg) was injected at 6.0 mL/s, and images were taken after 80 s with a rotation time of 0.5 s and pitch factor of 0.984. All images were reconstructed using a standard reconstruction algorithm with a slice thickness of 0.625 mm and reconstruction interval of 0.625 mm, and the FOV was sized to fit the patient.

### 2.2 Image processing and data analyses

**FIGURE 2:**
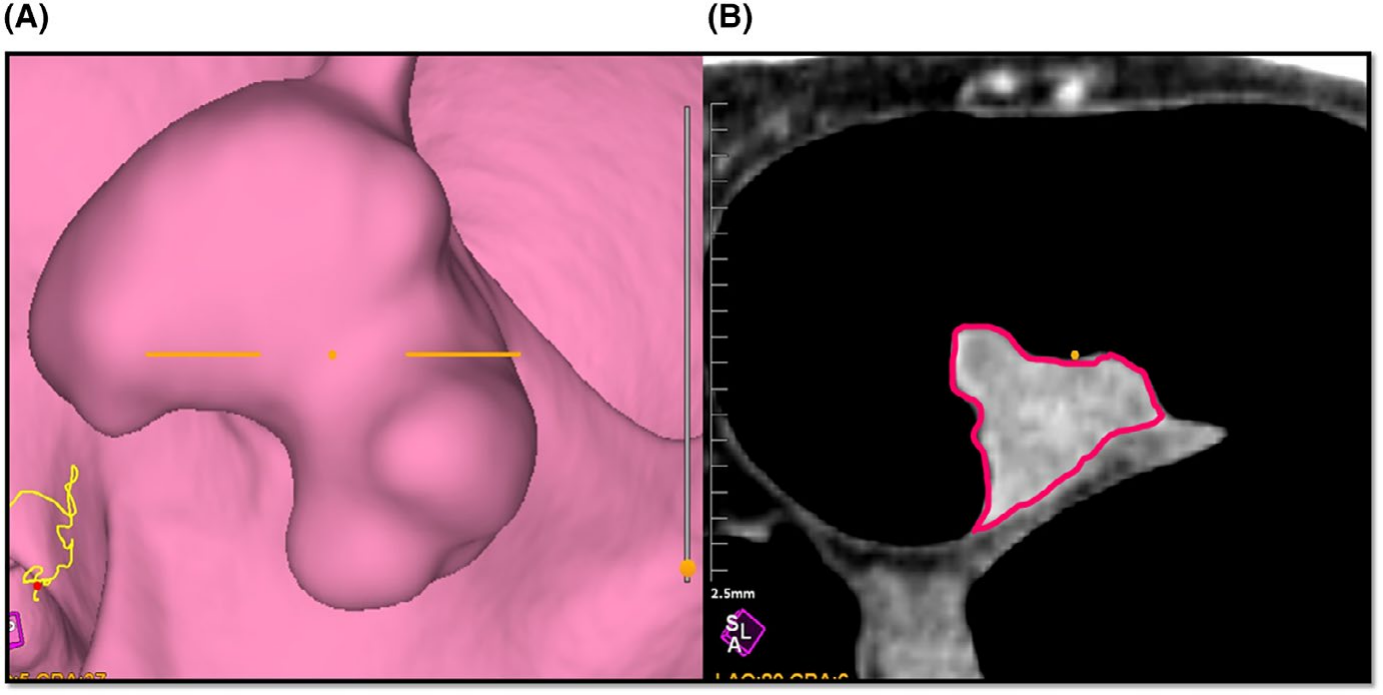
Texture parameter measurements. (A) The constructed virtual endoscopic image; the tumor site can be identified, and an arbitrary cross‐sectional view is selected. (B) Region of interest (ROI) was delineated around the peripheral boundary on the multiplanar reconstruction (MPR) image.

Imaging analyses were performed using the Attractive imaging analysis software program (PixSpace Ltd., Fukuoka, Japan). By importing the DICOM images of the portal phase CTC into the imaging analysis software program, a virtual endoscopic image was automatically constructed. The tumor site could then be identified from the constructed virtual endoscopic image, and an arbitrary cross‐sectional view could be selected. In this study, we manually drew a region of interest (ROI) from a multiplanar reconstruction (MPR) image of the same area according to the maximum diameter of the tumor (Figure 2).

Image analyses were performed by an independent observer (H.M., with 8 years of experience in CT interpretation).

Eight texture parameters, including the fractal dimension (FD), skewness, kurtosis, entropy, and gray‐level co‐occurrence matrix (GLCM) texture parameters, including GLCM‐correlation, GLCM‐autocorrelation, GLCM‐entropy, and GLCM‐homogeneity, in the ROI area were calculated. In addition, the correlation, autocorrelation, entropy, and homogeneity were evaluated as GLCM parameters.

We examined inter‐operator reproducibility using 20 patients' data which were randomly selected from 263 patients. Two observers (H.M. and T.T.) measured the parameters of tumors using the aforementioned techniques, and the intraclass correlation (ICC) was calculated. All the parameters (FD, skewness, kurtosis, entropy, and GLCM) had a value of 0.7 or higher.

#### 2.2.1 Fractal analysis

An FA is used to quantify the complexity of clinical images. The numerical value obtained with an FA is called the FD. The FD was measured using the box‐counting method^5^ and is defined by the following equation: $N_{L}$ = *KL* ^−FD^, where *L* is the box size, and $N_{L}$ is the number of boxes of size *L* required to cover the object. log *K* can be obtained from the *y*‐intercept obtained by linear regression of a log plot of $N_{L}$ vs. *L*.

#### 2.2.2 Histogram analyses

This study used three histogram parameters: skewness, kurtosis, and entropy. Skewness is a statistic that expresses if the distribution is symmetric or not. If a distribution is symmetric, statistical number is 0. If skewness is greater or less than 0, then it is called right‐skewed or left‐skewed and a distribution expresses asymmetric. Kurtosis is a statistic that tells us if a distribution is taller or shorter than a normal distribution. High kurtosis tends to have higher peak, in contrast low kurtosis tends to be flatter. Entropy is a statistic that heterogeneity, and is an indicator of the amount of information in an image. It becomes larger when pixels have much value of density.^6^

#### 2.2.3 GLCM

The GLCM is an efficient texture analysis method proposed by Haralick that uses second‐order statistics to characterize the properties of two or more pixel values occurring at a particular location.^7^ It is widely used as a powerful tool because of its ability to identify second‐order spatial relationships between pixels or voxels in input image data.^8^ The GLCM is a constructed matrix in which *P* (*i*, *j*) describes the probability of a pair of gray levels (*i* and *j*) occurring in an image. All gray‐level pairs are separated by a certain distance in a certain direction. In this study, GLCM was computed at a distance of 1 voxel, and the direction angles were 0, 45, 90, and 135. We took the average of the GLCM for the four directions as the texture feature values. The formulae for calculating the texture feature values in this study are as follows^9^:

$$\text{Correlation}=\sum_{i=1}^{G}\sum_{j=1}^{G}\frac{\left(i-\mu \right)\left(j-\mu \right)P\left(i,j\right)}{\sigma^{2}}$$

$$\text{Autocorrelation}=\sum_{i=1}^{G}\sum_{j=1}^{G}\mathrm{ijP}\left(i,j\right)$$

$$\text{Entropy}\left(H\right)=-\sum_{i=1}^{G}\sum_{j=1}^{G}P\left(i,j\right)\log_{2}\left[P\left(i,j\right)\right]$$

$$\text{Homogeneity}=\sum_{i=1}^{G}\sum_{j=1}^{G}\frac{P\left(i,j\right)}{1+{\left(i+j\right)}^{2}}$$

### 2.3 The pathological diagnosis

The histopathological evaluation of the surgical specimen was performed after surgery by board‐certified pathologists at our institute.

Tumor staging (T), nodal staging (N), and lymphatic and vascular invasion status of each specimen were evaluated. These histopathological features were assessed using the union for international cancer control staging (UICC) TNM classification of colorectal cancer.^10^

### 2.4 Statistical analyses

All statistical analyses were performed using the EZR software program (Jichi Medical University Saitama Medical Center, Saitama, Japan).^11^ Patients were divided into two groups according to the histological findings and the Mann–Whitney *U* test was applied for the comparison. The median value of the texture parameter was used as the cutoff value for the comparison with the overall survival (OS). A Kaplan–Meier analysis was performed for the OS, and the Cox regression test was used. We expressed hazard ratios (HRs) with 95% confidence intervals (CIs) using this model. *p* < 0.05 was considered statistically significant difference.

## 3 RESULTS

This study included 156 men and 107 women with a median age of 70 (range: 22–101) years old. The median tumor size was 35 (range: 3–130) mm. The patients' characteristics are listed in Table 1.

**TABLE 1:** Patient characteristics.

Patient demographics	Variables	Value
Age	Median (min–max)	70 (22–101)
Sex	Male/female	156/107
Localization of cancer	C/A/T/D/S/R	26/56/32/8/74/66
Tumor size of cancer (mm)	Median (min–max)	35 (3–130)
Pathological T stage	p T 1/2/3/4	59/38/100/65
Pathological N stage	p N 0/1/2/3	167/54/30/11
Lymphatic invasion	Ly −/+	207/56
Venous invasion	V −/+	95/168

Abbreviations: A, ascending colon; C, cecum; D, descending colon; R, rectum; S, sigmoid colon; T, transverse colon.

### 3.1 Associations of texture parameters with pathological factors

Associations between texture parameters and pathological features are shown in Table 2. All texture parameters except skewness showed significant differences between the pT1–2 and pT3–4 groups. GLCM‐homogeneity showed a significant difference between the N‐negative and N‐positive groups (*p* = 0.004). GLCM‐correlation and GLCM‐homogeneity levels showed significant differences between the Ly‐negative and Ly‐positive groups (*p* = 0.001 and 0.012, respectively). FD, GLCM‐entropy, GLCM‐correlation, and GLCM‐autocorrelation showed significant differences between the V‐negative and V‐positive groups (*p* = 0.001, 0.033, 0.021, and 0.046, respectively).

**TABLE 2:** Associations of texture parameters with clinicopathological features.

	Pathological T stage			Pathological N stage			Lymphatic invasion			Venous invasion		
	p T1–2	p T3–4	*p* Value	p N−	p N+	*p* Value	Ly−	Ly+	*p* Value	V−	V+	*p* Value
FD	1.93 (1.83 to 1.98)	1.92 (1.82 to 1.96)	0.001*	1.93 (1.83 to 1.98)	1.93 (1.82 to 1.96)	0.098	1.93 (1.83 to 1.98)	1.93 (1.82 to 1.93)	0.599	1.94 (1.82 to 1.98)	1.924 (1.83 to 1.96)	0.001*
Kurtosis	14.5 (1.82 to 59.7)	20.3 (2.92 to 120)	0.001*	16.7 (1.82 to 103)	19.6 (2.92 to 121)	0.478	17.6 (1.82 to 121)	19.9 (2.91 to 95.1)	0.535	15.8 (2.83 to 71.3)	19.5 (1.82 to 121)	0.115
Skewness	−2.27 (−5.87 to 0.89)	−2.84 (−7.24 to 2.39)	0.069	−2.46 (−7.2 to 1.61)	−2.56 (−7.24 to 2.39)	0.956	−2.47 (−7.24 to 2.39)	−2.62 (−7.19 to 1.19)	0.984	−2.33 (−6.71 to 0.89)	−2.67 (−7.24 to 2.39)	0.211
Entropy	6.73 (5.54 to 7.62)	6.92 (5.66 to 7.14)	0.001*	6.82 (5.54 to 8.21)	6.89 (6.03 to 7.76)	0.137	6.85 (5.54 to 8.21)	6.85 (6.03 to 7.76)	0.772	6.76 (5.54 to 7.47)	6.87 (5.60 to 8.21)	0.033*
GLCM correlation	0.69 (0.48 to 0.90)	0.73 (0.55 to 0.95)	0.001*	0.70 (0.48 to 0.95)	0.72 (0.57 to 0.94)	0.075	0.69 (0.48 to 0.95)	0.74 (0.55 to 0.94)	0.001*	0.70 (0.48 to 0.90)	0.72 (0.55 to 0.95)	0.021*
GLCM autocorrelation	39 419 (12 265 to 52 714)	42 439 (4966 to 56 015)	0.018*	40 645 (6080 to 53 350)	41 005 (4966 to 56 015)	0.829	40 838 (4966 to 53 935)	41 850 (10 121 to 56 015)	0.861	39 490 (4966 to 56 015)	42 207 (6080 to 54 061)	0.046*
GLCM entropy	10.4 (7.01 to 12.5)	10.6 (8.56 to 12.8)	0.008*	10.4 (7.01 to 12.8)	10.7 (8.56 to 12.7)	0.064	10.5 (7.01 to 12.8)	10.76 (8.56 to 12.5)	0.182	10.6 (7.01 to 12.5)	10.5 (8.56 to 12.8)	0.416
GLCM homogeneity	0.09 (0.02 to 0.21)	0.11 (0.04 to 0.33)	0.001*	0.09 (0.02 to 0.23)	0.11 (0.04 to 0.33)	0.004*	0.09 (0.01 to 0.26)	0.11 (0.05 to 0.33)	0.012*	0.09 (0.01 to 0.26)	0.10 (0.04 to 0.33)	0.064*

Abbreviations: FD, fractal dimension; GLCM, gray‐level co‐occurrence matrix.

* *p* < 0.05.

### 3.2 Correlations of texture parameters with the OS

The median value of the texture parameter was used as cut off, and the population was divided into two groups using the median of the texture parameters as the cutoff value.

**FIGURE 3:**
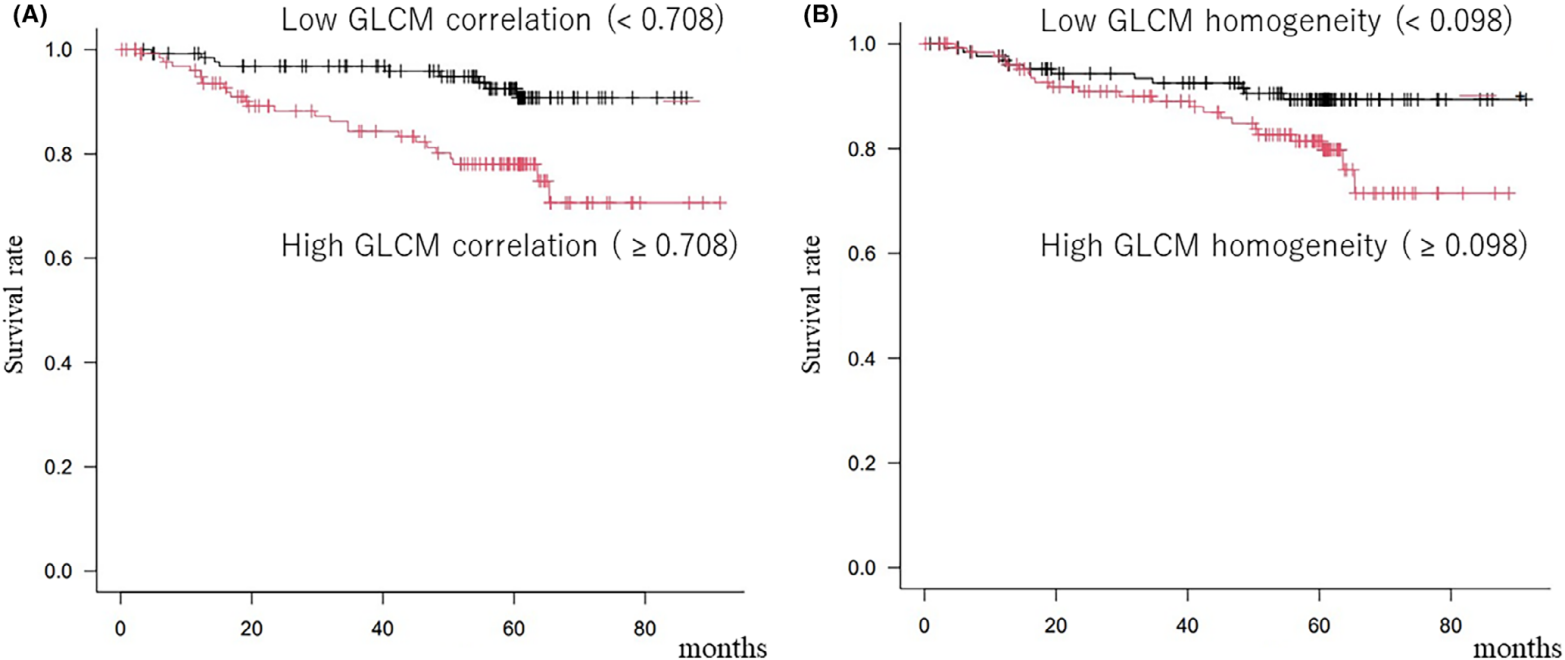
Relationship of texture parameters with the overall survival. (A) The Kaplan–Meier curve demonstrated that cases with high GLCM‐correlation (GC) tended to have a worse overall survival than others (*p* = 0.001). (B) The Kaplan–Meier curve demonstrated that cases with high GLCM‐homogeneity (GH) tended to have a worse overall survival than others (*p* = 0.02).

In the comparison of overall survival, GLCM‐correlation and GLCM‐homogeneity showed significant differences (Table 3). In the Kaplan–Meier analysis (Figure 3), patients with high GC (≥0.708) tumors or those with high GLCM‐homogeneity tumors (≥0.098) showed a significantly worse OS than others (*p* = 0.001 and 0.04, respectively). Table 4 shows the results of univariate and multivariate analyses of tumor parameters for the OS. A univariate analysis using Cox's regression model showed that the tumor depth, lymph node metastasis, venous invasion, GLCM‐correlation, and GLCM‐homogeneity were significantly correlated with the OS. A multivariate analysis demonstrated that lymph node metastasis and GLCM‐correlation were independent prognostic factors for the OS (*p* = 0.001 and 0.021, respectively).

**TABLE 3:** Association of texture parameters for overall survival.

Texture parameters	HR	95% CI	*p*
FD	1.306	0.668–2.551	0.433
Kurtosis	1.161	0.597–2.260	0.659
Skewness	1.048	0.540–2.036	0.890
Entropy	1.216	0.625–2.366	0.565
GLCM correlation	3.211	1.504–6.857	0.001*
GLCM autocorrelation	0.912	0.470–1.771	0.786
GLCM entropy	1.729	0.871–3.434	0.113
GLCM homogeneity	2.043	1.016–4.107	0.041*

*Note*: Cox's regression analysis. ※ cut off = median.

Abbreviation: GLCM, gray‐level co‐occurrence matrix.

* *p* Value < 0.05.

**TABLE 4:** Univariate and multivariate analyses of clinicopathological characteristics and texture parameters for overall survival.

	Univariate	Multivariate		
	*p* Value	HR	95% CI	*p* Value
GLCM correlation	0.041*	2.51	1.15–5.50	0.021*
GLCM homogeneity	0.001*	1.07	0.52–2.21	0.859
p T1–2 vs. p T3–4	0.001*	2.48	0.71–8.66	0.154
p N− vs. p N+	0.001*	4.82	2.14–10.87	0.001*
V− vs. V+	0.001*	3.30	0.98–11.07	0.053

*Note*: Cox's regression analysis.

Abbreviation: GLCM, gray‐level co‐occurrence matrix.

* *p* Value < 0.05.

GLCM‐correlation and GLCM‐homogeneity were included in the multivariate analysis, but no correlation was found (correlation coefficient = 0.309).

## 4 DISCUSSION

In this study, we used the term “malignancy” as prognosis of the tumor. Malignant tumors generally have structural heterogeneity, which is recognized as an important issue leading to malignancy of cancer and chemotherapy resistance. Hayano et al.^12^ reported that structural heterogeneity leads to a heterogeneous blood supply within the tumor, which may result in a hypoxic tumor environment. They further found that hypoxia and necrosis contribute to intratumoral heterogeneity by increasing the number of low‐density areas within the tumor. Therefore, intratumoral heterogeneity may be associated with treatment response and the identification of drug targets, and survival. Thus, quantification of structural abnormality of the tumor has a potential to be a biomarker for cancer treatment. With the wide availability of CT examinations in clinical practice, the analysis of tumor structural heterogeneity on CT images is expected to become more useful and practical as a biomarker for cancer.

Several previous reports have shown that heterogeneity of tumor structures on CT correlates with the treatment response and prognosis in head and neck cancer, esophageal cancer, lung cancer, and renal cell carcinoma.^13^, ^14^, ^15^, ^16^, ^17^, ^18^, ^19^ In the present study, we measured the heterogeneity of CE‐CTC images using a texture analysis, which is useful for quantifying the complexity, direction, and contrast variation in digital images. A higher GLCM measured on CE‐CTC images was significantly associated with malignancy of CRC and a worse prognosis. GLCM‐correlation was shown to be an independent prognostic factor for the OS in a multivariate analysis. This suggests that GLCM may be associated with intratumoral heterogeneity. Intratumoral heterogeneity is multifactorial and has been reported to be related to several factors, including hypoxia, necrosis, angiogenesis, and genetic variation.^20^, ^21^

Bum et al.^22^ reported that GLCM is a strong predictor of the OS in pancreatic cancer, and Chen et al.^23^ reported that GLCM may be a predictor of OS in small‐cell lung cancer. However, there are currently no reports showing an association between GLCM and long‐term outcome of the patients with CRC. As mentioned above, in the current practice of CRC, the evaluation of malignancy is determined by pathological factors. There are few useful biomarkers that can assess the malignancy and the prognosis of cancer before surgery, such as tumor differentiation, tumor depth, lymph node metastasis, and distant metastasis. We therefore believe that if GLCM becomes possible to predict malignancy of cancer before surgery, appropriate treatment may be selected.

The present study used preoperative CE‐CTC images, which are relatively easy to obtain and differ little between centers. CTC is useful in the preoperative examination of CRC because it is less strongly affected by patient factors, such as body size, bowel shape, and length, than colonoscopy and can evaluate the proximal bowel even in cases of severe stenosis where the endoscope cannot pass through. In the present study, GLCM, which is a texture parameter obtained using CE‐CTC images, was suggested as a new biomarker for detecting and stratifying malignancy of CRC.

However, our study had several limitations. First, it used single‐center, retrospective data. Therefore, the findings should be confirmed through multicenter prospective investigations. Second, the method of tumor ROI delineation was subjective and performed by a single team leader. Therefore, a new, reproducible, and reliable tumor segmentation method is required. Third, our analysis used CE‐CTC images; however, a volumetric analysis may be more representative of the tumor structure. Therefore, a three‐dimensional analysis approach should be developed.

## 5 CONCLUSION

GLCM is the only biomarker correlated with prognosis that can be measured preoperatively for colorectal cancer. GLCM derived from texture analyses using CE‐CTC images may become a potentially viable biomarker for assessing malignancy of CRC before surgery.

## 6 AUTHOR CONTRIBUTIONS

Hisashi Mamiya: Writing – original draft preparation; Formal analysis and investigation; Conceptualization; visualization; data curation. Toru Tochigi: Writing – review and editing; conceptualization; Methodology; Funding acquisition; Resources; project administration. Koichi Hayano: Writing – review and editing; conceptualization; Methodology; Supervision; project administration. Gaku Ohira: Supervision; conceptualization; validation. Shunsuke Imanishi: Supervision; conceptualization. Tetsuro Maruyama: Supervision; conceptualization; data curation. Yoshihiro Kurata: Supervision; conceptualization. Yumiko Takahashi: Supervision; conceptualization. Atsushi Hirata: Supervision; conceptualization. Hisahiro Matsubara: Supervision; conceptualization.

## FUNDING INFORMATION

There are no relevant financial or nonfinancial relationships to disclose.

## CONFLICT OF INTEREST STATEMENT

Prof. Matsubara is a member of the Editorial Board for *AGSurg*.

## ETHICS STATEMENT

Approval of the research protocol: N/A.

Informed Consent: All patients provided their written informed consent to undergo a contrast‐enhanced CTC examination, but participation was not required because of the retrospective nature of this study.

Registry and the Registration No. of the study/trial: N/A.

Animal Studies: N/A.
